# Deep learning-based prediction for time-dependent chloride penetration in concrete exposed to coastal environment

**DOI:** 10.1016/j.heliyon.2023.e16869

**Published:** 2023-06-01

**Authors:** Lingjie Wu, Weiqiang Wang, Chenchi Jiang

**Affiliations:** a College of Civil Engineering and Architecture, Wenzhou University, Wenzhou 325035, China; b Key Laboratory of Engineering and Technology for Soft Soil Foundation and Tideland Reclamation of Zhejiang Province, Wenzhou 325035, China; c Wenzhou Engineering Technical Research Center on Building Energy Conservation and Emission Reduction & Disaster Prevention and Mitigation, Wenzhou, Zhejiang, 325035, China; d Zhejiang Collaborative Innovation Center of Tideland Reclamation and Ecological Protection, Wenzhou, Zhejiang, 325035, China

**Keywords:** Deep learning, Concrete, Chloride, Prediction

## Abstract

The application of deep learning methods in civil engineering has gained significant attention, but its usage in studying chloride penetration in concrete is still in its early stages. This research paper focuses on predicting and analyzing chloride profiles using deep learning methods based on measured data from concrete exposed for 600 days in a coastal environment. The study reveals that Bidirectional Long Short-Term Memory (Bi-LSTM) and Convolutional Neural Network (CNN) models exhibit rapid convergence during the training stage, but fail to achieve satisfactory accuracy when predicting chloride profiles. Additionally, the Gate Recurrent Unit (GRU) model proves to be more efficient than the Long Short-Term Memory (LSTM) model, but its prediction accuracy falls short compared to LSTM for further predictions. However, by optimizing the LSTM model through parameters such as the dropout layer, hidden units, iteration times, and initial learning rate, significant improvements are achieved. The mean absolute error (MAE), determinable coefficient (*R*^2^), root mean square error (RMSE), and mean absolute percentage error (MAPE) values are reported as 0.0271, 0.9752, 0.0357, and 5.41%, respectively. Furthermore, the study successfully predicts desirable chloride profiles of concrete specimens at 720 days using the optimized LSTM model.

## 1 Introduction

Chloride corrosion is one of the key factors affecting the concrete durability in coastal and even inland saline-alkali areas [1,2]. Corrosion of reinforcement induces cracking of concrete cover, which in turn further accelerates the rebar corrosion and ultimately affects the durability, applicability, and safety of the concrete structure.

Currently, research on chloride penetration in concrete primarily focuses on experimental studies and finite element simulation. With the increasing accumulation of experimental data, machine learning methods have become widely utilized for data analysis and prediction. Some commonly employed methods include Support Vector Machine (SVM) and Artificial Neural Network (ANN) [[3], [4], [5], [6], [7], [8], [9], [10], [11]]. Zhu et al. [7] conducted an in-depth analysis of critical chloride concentration using the ANN method by summarizing and organizing published literature data. Yu [9] analyzed the impact of water-cement ratio, concrete cover depth, coarse aggregate volume, and ambient temperature and humidity on chloride diffusion using machine learning methods such as decision tree (DT), linear regression (LR), Back Propagation (BP) neural network, random forest (RF), and ridge regression (RR). Song and Kwon [10] developed a neural network model considering eight input parameters, including water-cement ratio and mineral admixture, and successfully predicted chloride diffusion coefficients with an average difference of approximately 7.5% compared to measured values. Delnavaz and Ramezanianpour [11] investigated the impact of water-binder ratio, silica fume content, and carbonation degree on chloride diffusion in concrete using an ANN model. Tran et al. [12] employed gradient boosting (GB), ANN, and RF models to predict chloride content in concrete, all of which demonstrated good performance and achieved satisfactory prediction accuracy. Hoang et al. [13] utilized multivariate adaptive regression spline (MARS) and multi-gene genetic programming (MGGP) to establish a machine learning model for chloride ion diffusion prediction. They compared the results with those obtained from ANN and least squares support vector regression, and found that MARS exhibited the best prediction performance. Gao et al. [14] proposed a novel method for predicting the service life of tunnel structures using genetic programming (GP) and actual engineering data. Their results indicated that the GP method offered superior computational efficiency compared to the ANN model.

However, the traditional machine learning methods are usually shallow machine learning models, which are not applicable to solve multivariate complex issues, hard to extract parameters and characteristics, difficult to express functional relationship, and produce prediction results subjected to working and environmental conditions [15]. ANN and BP methods require a high quantity and quality of data, and are prone to getting stuck in local optima. They are also sensitive to initialization and hyperparameters. As for GP methods, they face challenges such as high computational cost and lack of global optimization guarantees. Deep learning, as a prominent branch of machine learning, addresses these limitations by introducing deep neural networks with enhanced hidden layers that incorporate both feedforward and feedback connections. Additionally, deep learning models incorporate memory functions, resulting in improved applicability for data analysis and prediction [16]. In recent years, with advancements in neural network technology, deep learning has emerged as a research hotspot in various industries. In the field of civil engineering, its applications primarily focus on damage identification and prediction of deformation and settlement [[17], [18], [19], [20], [21], [22], [23], [24]]. For instance, Yang et al. [21] utilized both statistical and deep learning models to predict deformation in a concrete dam based on actual detection data. Their findings recommended the Long Short-Term Memory (LSTM) model when sufficient training data is available, while the least squares regression method is recommended when training data is limited to achieve satisfactory prediction results. Qu et al. [22] also analyzed deformation in a concrete dam using the LSTM method. Tra et al. [23] employed LSTM, Gate Recurrent Unit (GRU), and Recurrent Neural Network (RNN) methods to predict and analyze the remaining service life of reinforced concrete beams, demonstrating that the LSTM method achieved superior prediction accuracy. Based on the detection data of 3,368 bridges, Miao et al. [24] established an LSTM model to analyze the effects of 12 potential influencing factors on bridge cracking, which proved that the LSTM model could help obtain satisfactory prediction accuracy, and the predicted degradation curve of bridge performance also provided reference for subsequent maintenance of the bridges.

However, the using of deep learning to study chloride transport in concrete just starts not long before [25,26]. Ramani et al. [25] identified the rebar corrosion by deep learning method and predicted the cracking time of concrete cover on this basis. Shin et al. [26] employed the Convolutional Neural Network (CNN) model to identify images of the outer surface of concrete specimens subjected to different water-cement ratios. They further aimed to analyze these images to predict the corresponding chloride diffusion coefficient.

The aforementioned discussions highlight the growing utilization of deep learning methods in civil engineering research. However, their application in the study of chloride transport in concrete, specifically in the context of time-series prediction, is still in its early stages. The chloride profile exhibits highly nonlinear behavior and significant time-dependent characteristics, making deep learning methods a suitable choice for time-series prediction in this area. In light of this, this paper employs various deep learning methods such as CNN, LSTM, GRU, and Bi-LSTM to predict and analyze chloride profiles using measured data from concrete exposed to a coastal environment for 600 days. The results are then compared with those obtained using traditional machine learning methods like BP and SVM. The findings of this study contribute valuable insights into the application of deep learning methods for studying chloride transport in concrete, both in terms of theoretical advancements and practical implications.

## 2 Theoretical backgrounds

### 2.1 Deep-learning model

**Fig. 1:**
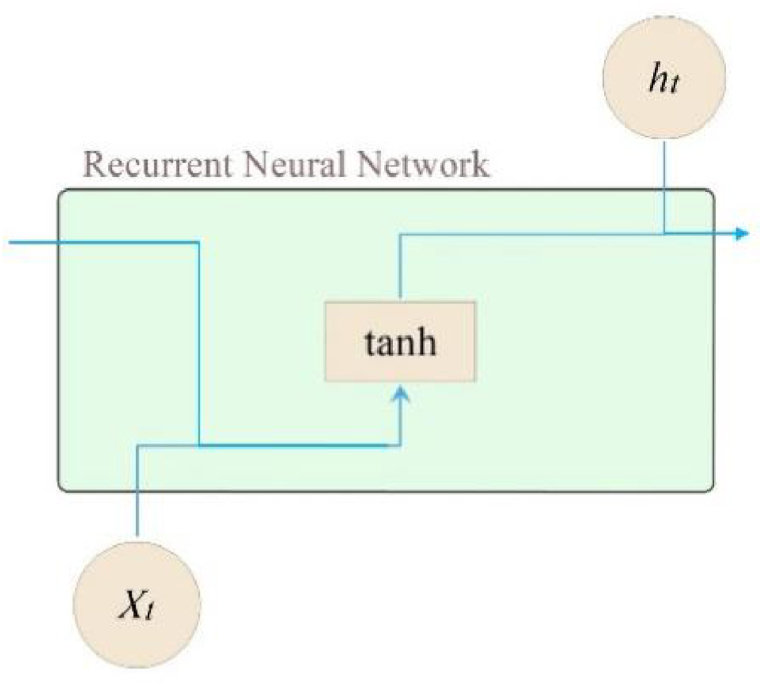
RNN structure diagram.

RNN, which stands for Recurrent Neural Network, is a type of neural network specifically designed for processing sequential data. It takes sequences as input and performs recurrent computations along the evolution direction of the sequence. RNNs are characterized by their chain-like structure, where all nodes (referred to as recurrent units) are interconnected, as depicted in Fig. 1. This architecture enables RNNs to effectively handle multivariate time-series data with varying lengths. The input information of each layer relies on the output information of previous layer, so that the output information of previous and former layers could possibly affect the current output information. However, researchers have found that, the adoption of RNN may lead to gradient disappearance or explosion problems when processing long-term data [16,23]. Therefore, a LSTM network is proposed, which judges whether or not to save current data by adding three gates. It helps to eliminate the invalid data and achieve long-distance transmission of key information existed in early data. With LSTM, it is easier to extract the potential long-term dependent information [27].

**Fig. 2:**
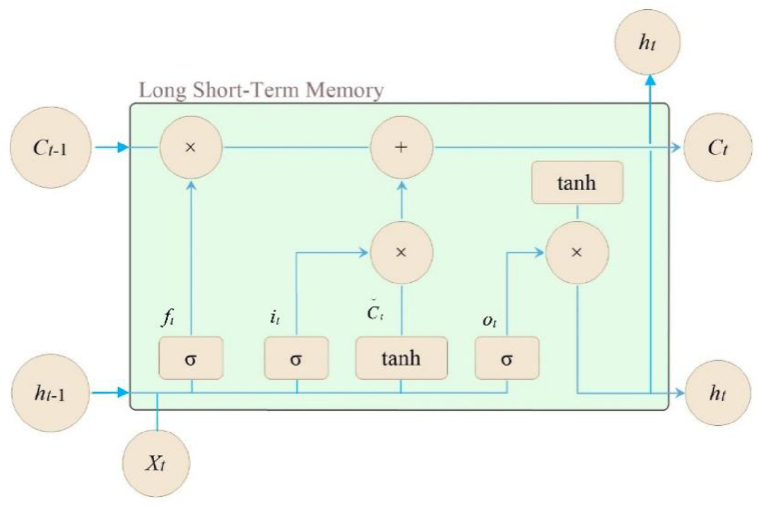
LSTM structure diagram.

Fig. 2 illustrates the structure of a LSTM network. In this figure, several components are depicted: *f*_t_ represents the forget gate at time t; *i*_t_ represents the input gate at time t; *o*_t_ represents the output gate at time t; *C*_t_ represents the cell unit state at time t; ${\tilde{C}}_{t}$ represents the temporary cell unit state at time t; *h*_t_ represents the hidden layer information at time t. *X* represents the input information at time t; tanh represents the tangent function; and *σ* represents the Sigmoid function [27]. Mathematical expressions of all parameters are shown by Eqs. (1), (2), (3), (4), (5), (6) [27].

(1) $$f_{t}=\sigma \left(W_{f}\left[h_{t-1},x_{t}\right]+b_{f}\right)$$

(2) $$i_{t}=\sigma \left(W_{i}\left[h_{t-1},x_{t}\right]+b_{i}\right)$$

(3) $${\tilde{C}}_{t}=\tanh\left(W_{c}\left[h_{t-1},x_{t}\right]+b_{c}\right)$$

(4) $$C_{t}=f_{t}⊗C_{t-1}+i_{t}⊗{\tilde{C}}_{t-1}$$

(5) $$o_{t}=\sigma \left(W_{o}\left[h_{t-1},x_{t}\right]+b_{o}\right)$$

(6) $$h_{t}=o_{t}\;\tanh⊗C_{t}t$$

where ⊗ is multiplication of vectors; *W*_*f*_, *W*_*i*_, *W*_*c*_ and *W*_*o*_ are weight matrices of neural networks; *b*_*f*_, *b*_*i*_, *b*_*c*_ and *b*_*o*_ are bias vectors.

**Fig. 3:**
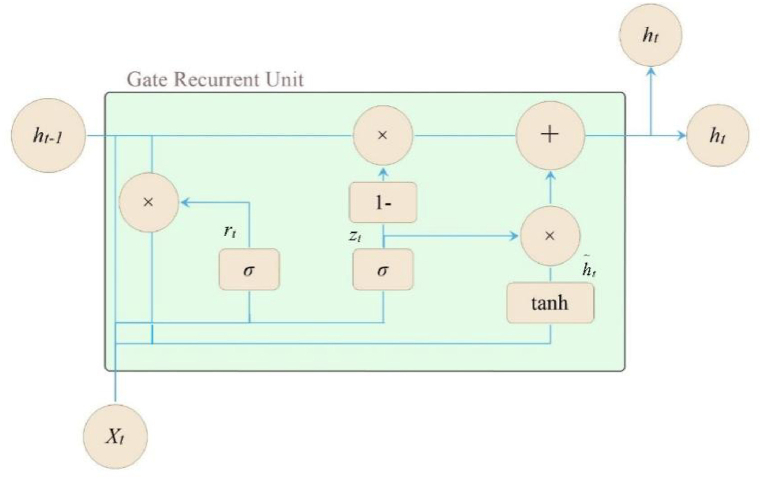
GRU structure diagram.

GRU is a variant network of LSTM. Compared with LSTM, GRU network merges unit states and outputs into hidden states, and transmits information through the hidden states. In addition, GRU integrates the forget gate and input gate originally existed in LSTM into an update gate [28]. The number of parameters used in GRU network is reduced by one third compared to the number used in LSTM. Fig. 3 illustrates the structure of a GRU network.

### 2.2 Chloride profiles prediction based on deep learning method

**Fig. 4:**
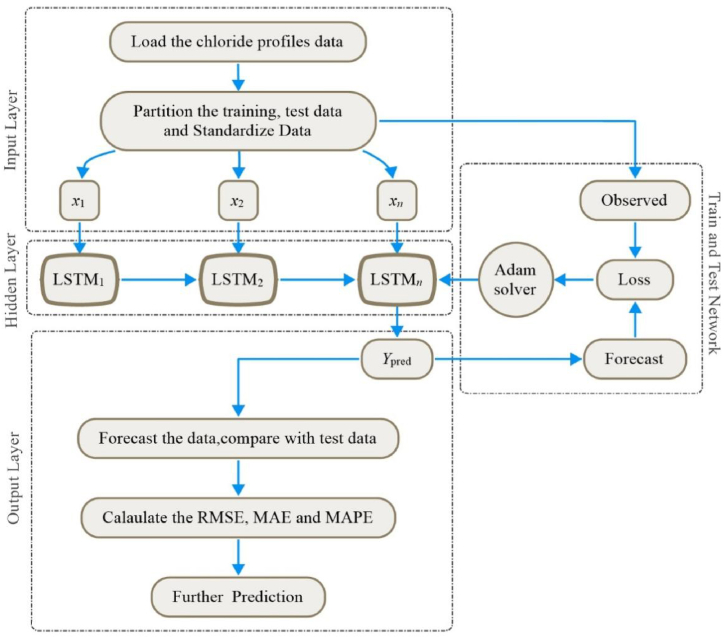
Chloride profiles forecast by LSTM model.

Based on the above-mentioned deep learning models, this work taking LSTM model as an example, relative time-series prediction model is built up combining with the measured chloride profiles data. Fig. 4 shows the overall framework of the model, including modules of input layer, hidden layer, training network and output layers. The input layer is responsible for preprocessing the original data to satisfy the requirements on network input; the output layer provides the prediction results; and the network training is achieved by ADAM solver.

By the dynamic adjustment of long short-term memory network, the model can fully learn the nonlinear correlation in different time-series, thus capturing the time-dependent mechanism of chloride transport in concrete. This method can not only lower the requirement on diachronic data quality in the prediction process, but also improve the prediction accuracy and interpretability essentially.

In the input layer, data need to be split into training set and test set. In addition, in order to better fit and prevent training divergence, data must be normalized as well. For sample data *x*, its normalized calculation formula is given by Eq. (7):

(7) $$x^{*}=\frac{x-\min\left(x\right)}{\max\left(x\right)-\min\left(x\right)}$$

where max(*x*) is the maximum value, and min(*x*) is the minimum value.

In the hidden layer, an LSTM layer with 128 hidden units is used by default, while the Adam solver is taken to conduct the neural network training. The number of hidden units determines the volume of information learned in this layer. More hidden units used in the training can produce more accurate results, but it can also possibly lead to overfitting of training data [29].

In the output layer, to evaluate the prediction accuracy of the model, four commonly used indicators are employed to assess the model's performance. These indicators include the mean absolute error (MAE), mean absolute percentage error (MAPE), root mean square error (RMSE), and determinable coefficient (R^2^). The calculation formulas for these indicators are provided in Eqs. (8), (9), (10), (11) as shown below [29].

(8) $$\text{MAE}=\frac{1}{n}\sum_{i=1}^{n}\left|{Y_{Pred}}_{,i}-Y_{Test,i}\right|$$

(9) $$\text{MAPE}=\frac{1}{n}\sum_{i=1}^{n}\left|\frac{Y_{Pred,i}-Y_{Test,i}}{Y_{Test,i}}\right|$$

(10) $$RMSE=\sqrt{\frac{1}{n}\sum_{i=1}^{n}{\left(Y_{Pred,i}-Y_{Test,i}\right)}^{2}}$$

(11) $$R^{2}=1-\frac{\sum_{i=0}^{n}{\left(Y_{Pred,i}-Y_{Test,i}\right)}^{2}}{\sum_{i=0}^{n}{\left(Y_{Test,i}-\bar{Y_{Test}}\right)}^{2}}$$

where *n* is the number of samples, *Y*_*Pred*_ is the predicted value of neural network, and *Y*_*Test*_ is the measured value.

MAE represents the average value of the absolute errors, providing a clear indication of the actual magnitude of the prediction errors. Its value ranges from 0 to infinity, with smaller values indicating lower model errors and better accuracy. RMSE is the arithmetic square root of the mean squared errors. It is used to assess the overall fitting quality of the model and its predictive accuracy. RMSE also ranges from 0 to infinity, and smaller values indicate better model fitting and higher accuracy. MAPE is a relative measurement that scales the MAE as a percentage of the actual values. It allows for comparison across different scales or variables. A smaller MAPE value indicates a higher level of accuracy in the predictions made by the model. *R*^2^ is the ratio between the explained variation and the total variation in a model. A higher *R*^2^ value implies that the portion of the variation explained by the model is greater, indicating a better fit of the model.

## 3 Experimental verifications

### 3.1 Chloride corrosion test and data collection

The chloride corrosion test is conducted in the tidal zone of a coastal terminal in Dinghai New City of Zhoushan, China. According to the meteorological and hydrological data of the investigation scene that, this area is characterized with about 20 °C average annual temperature, about 79% average annual humidity, and about 4.5 h per day being immersed in seawater every day. According to the detection and analysis of water sampled from the site, the content of free chloride in the seawater in this area is about 1.3%.

**Fig. 5:**
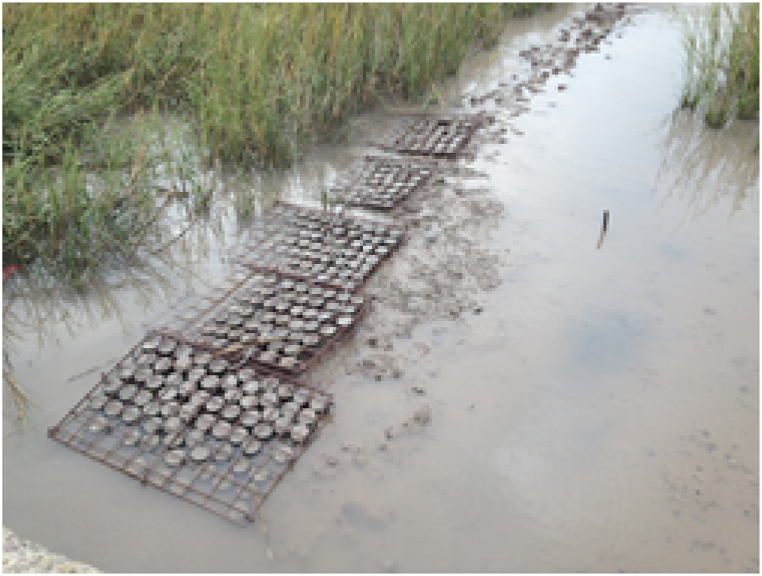
Field exposure of concrete specimens.

P•O32.5 R cement is used in this study to make cylindrical concrete specimens sizedφ100 mm × 50 mm with 0.45 water-cement ratio. Six specimens are prepared for each group. Fig. 5 shows the field exposure environment of the concrete specimens. The exposure time of concrete specimens are 120d, 240d, 360d, 480d and 600d, respectively. After that, the concrete grinder is adopted to grind inwards from the specimen surface and perform sampling every 2 mm. Meanwhile the American Thermo 720A acidimeter is applied to detect the chloride concentration in concrete (the mass percentage occupied in concrete).

### 3.2 LSTM model application

**Fig. 6:**
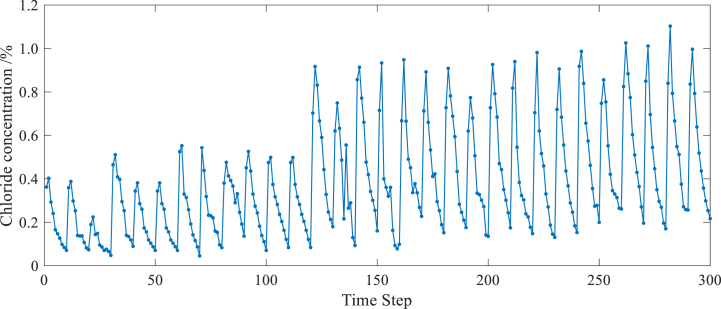
Observed data.

A total of 300 test data were obtained from the on-site experiment, as shown in Fig. 6. 90% of the test data, which is 270 measured chloride concentration values, were selected for training the network model, while the remaining 10% of the test data, which is 30 measured chloride concentration values, were used for testing and validation.

Relevant calculation parameters of LSTM model are as follows: the hidden unit is 128, the dropout layer is 0, the number of iterations is set to be 300, the initial learning rate is set to be 0.001, and other parameters are the default values of the software.

Since the test concrete has only one water-cement ratio, the sampling time interval is set to be a fixed period of 120d, and the specimen numbers of all groups are the same: each group contains 6 specimens. Therefore, in time-series prediction, the chloride concentration can be used as the only input variable and output variable in this model.

**Fig. 7:**
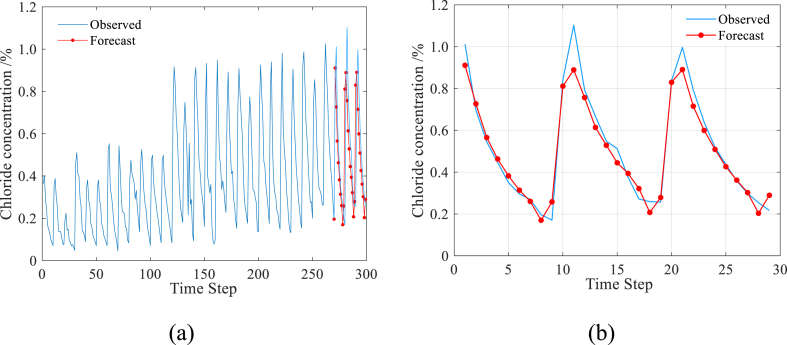
Prediction result by LSTM model.

In Fig. 7(a, b), the prediction results of the LSTM model are displayed. Fig. 7(b) demonstrates that the predicted values closely match the measured values, with only 0.049 MAE, 12.73% MAPE, 0.0607 RMSE, and 0.9457 R2 observed between the measured and predicted values. These metrics indicate a high level of prediction accuracy achieved by the LSTM model. Based on these results, it can be concluded that the LSTM model is well-suited for predicting the chloride transport in concrete exposed to a coastal environment.

**Fig. 8:**
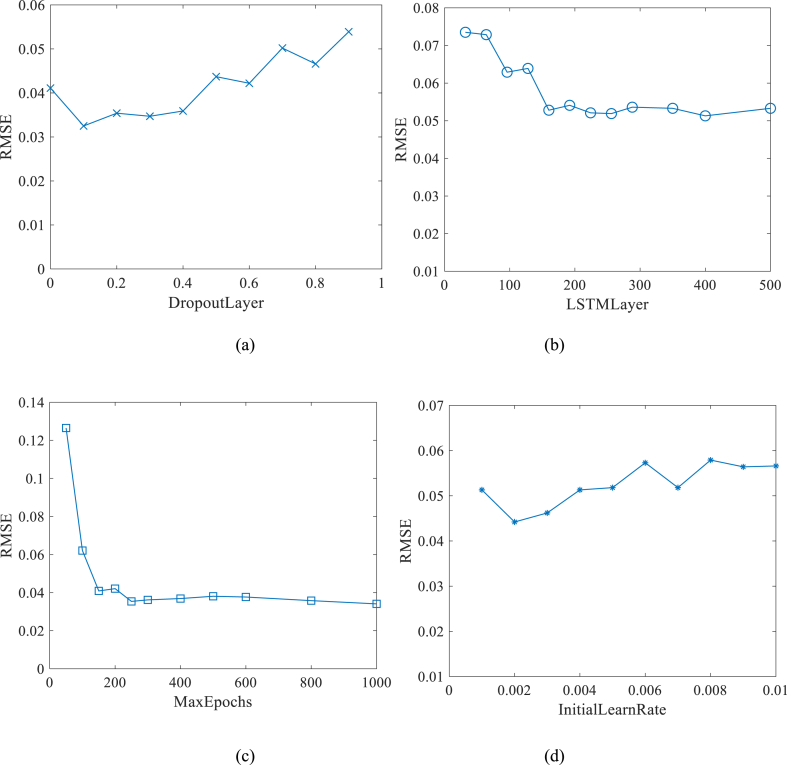
RMSE influencing factors.

Earlier studies have shown that, no matter for traditional machine learning method or the deep learning method, the setting of model parameters has significant effects on prediction accuracy [[30], [31], [32], [33]]. With sufficient times of iteration, most parameter optimization targeted at LSTM model focuses on the hidden unit and initial learning rate [32,33]. However, by further analyzing the model parameters that, the dropout layer may also affect the prediction accuracy of LSTM model. Based on the prediction results of LSTM model, Fig. 8 shows the effect of dropout layer, hidden unit, iterations, and initial learning rate on the RMSE.

**Fig. 9:**
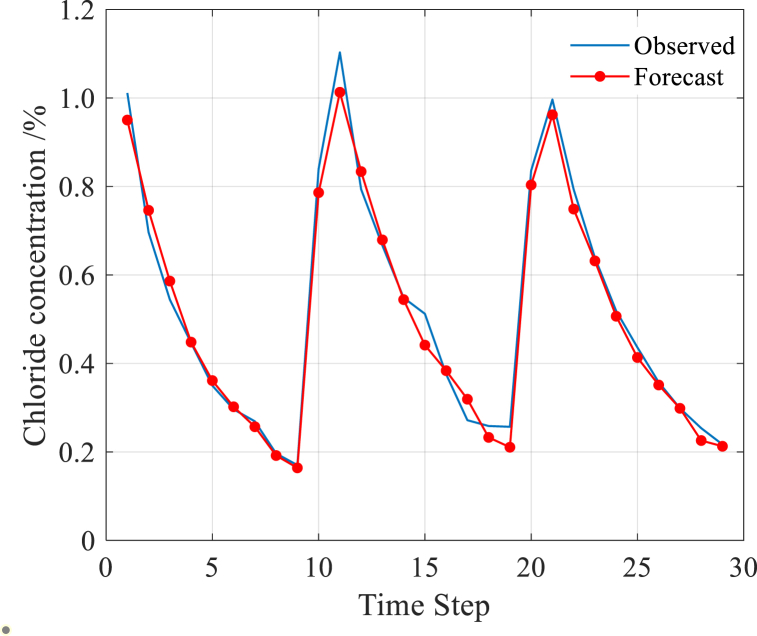
Prediction result by optimized LSTM model.

According to Fig. 8(a–d), the effect of the dropout layer on the RMSE value follows a pattern of initially decreasing and then increasing. The optimal prediction performance of the LSTM model is observed when the dropout layer is set to 0.1. Similarly, the initial learning rate also exhibits a similar effect on the RMSE value, with the optimal value found to be 0.002. In addition, the RMSE value decreases as the number of hidden units and iterations increases, stabilizing when the number of iterations exceeds 300 or the number of hidden units exceeds 192. Based on these findings, further optimization of the LSTM model parameters can be conducted. Numerical experiments were performed by setting the dropout layer to 0.1, initial learning rate to 0.002, hidden units to 192, and iterations to 500. The optimized prediction results are presented in Fig. 9.

Compared Figs. 9 and 7(b), the parameter optimization greatly improves the LSTM prediction accuracy, and makes the predicted value approach the measured value closely. On the other hand, after the parameter optimization, the values of four indexes for model evaluation change dramatically: MAE drops from 0.049 to 0.0271, MAPE reduces from 12.73% to 5.41%, RMSE declines from 0.0607 to 0.0357 and *R*^2^ rises from 0.9457 to 0.9752.

**Fig. 10:**
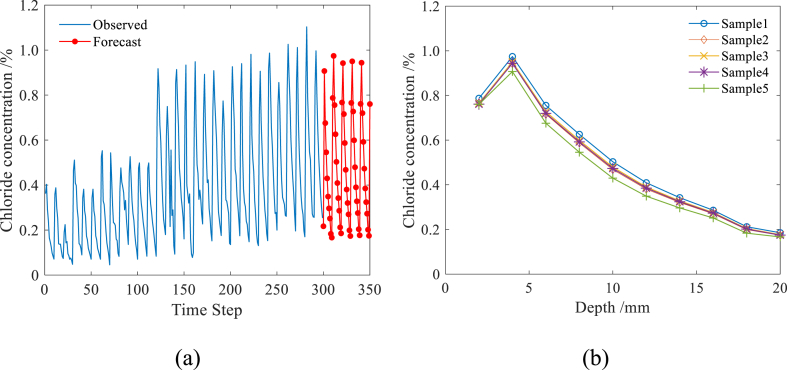
Chloride profiles predicted by optimized LSTM model.

**Fig. 11:**
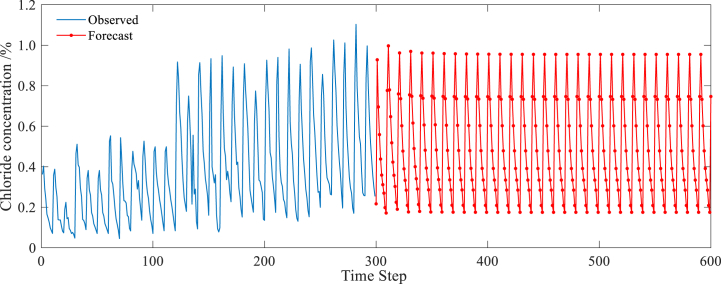
Chloride profiles predicted by optimized LSTM model with long time step.

Compared to traditional machine learning methods, the well-trained LSTM model has the advantage of making direct predictions for future time steps without requiring additional input data. Fig. 10(a, b) illustrates a portion of the chloride profiles of concrete specimens predicted by the LSTM model for 50 time steps. As shown in Fig. 10(a, b), the predicted chloride profiles of concrete specimens experiencing 720 days of corrosion align well with the actual chloride transport in the concrete. The measured and predicted chloride profiles are then fitted using Fick's second diffusion law, resulting in the corresponding apparent chloride diffusion coefficients at 240–720 days: 1.805, 1.578, 1.24, 1.064, 0.8196, and 0.6805 × 10^−12^, respectively. These findings indicate that the apparent chloride diffusion coefficient exhibits significant time-varying attenuation characteristics as the exposure time increases. This observation aligns with previous research results [[34], [35], [36], [37]], which highlight the pronounced time-dependent behavior of chloride transport in concrete. By further analyzing the time-dependent characteristics of the apparent chloride diffusion coefficient, an age factor of 0.4012 is obtained. In the context of a dry-wet cycle environment, literature often recommends age factors ranging from 0.2 to 0.55 [36]. Therefore, the obtained age factor of 0.4012 falls within the typical range suggested in existing research, which further improves the credibility of the prediction results obtained by LSTM model. Therefore, it is feasible to predict the chloride profiles in concrete exposed to coastal environment based on LSTM model, and the short-term prediction results are credible. It should be pointed out that, as a common defect of neural networks, with the increase of the prediction time step, the prediction accuracy will decrease due to the lack of measured data to update the trained network. As shown in Fig. 11, when the prediction time steps exceed 60, the chloride profiles predicted by the LSTM model has become stable, which is obviously inconsistent with the actual situation.

### 3.3 Model comparison and analysis

To conduct a comprehensive analysis of deep learning and traditional machine learning methods for predicting time-dependent chloride transport in concrete, several models were selected for numerical experiments. The models include CNN, GRU, Bi-LSTM, LSTM, BP, and SVM. To ensure a fair comparison and prevent parameter optimization from influencing the prediction accuracy, the main calculation parameters of each model were set to their default values. The specific parameters for each model are as follows:

(1) The hidden layer activation function of BP neural network is set as tansig, the trainlm is used as the training algorithm, the error control rate is 0.000001, and the learning coefficient is 0.01.
(2) The penalty coefficient c of SVM model is 4.0, and the gamma value is 0.8;
(3) The hidden unit of the LSTM model is 128, the Dropout layer is 0, the number of iterations is set to be 300, the initial learning rate is set to be 0.001, and the Adam is used as solver;
(4) The parameters of GRU and Bi-LSTM models are the same as those of the LSTM model;
(5) The number of convolution kernels in CNN model is 32, the size of convolution kernels is 3, and other parameters are the same as those of the LSTM model.

Table 1 presents the results of evaluation indexes for various prediction models. Among the commonly used neural network algorithms for time-series research, the GRU model demonstrates superior performance in terms of RMSE, MAE, MAPE, and *R*^2^. It achieves more accurate results compared to other models. Notably, the R2 value of the GRU model is 0.9721, significantly higher than other models, while the RMSE, MAE, and MAPE values are considerably lower. The LSTM model also shows favorable experimental results when compared to traditional BP and SVM models. The RMSE and MAE values of the LSTM model are lower than those of the BP and SVM models, and it attains an R2 value of 0.9457, which surpasses the BP and SVM models. This further validates the superiority of deep learning methods in time-series prediction. However, the prediction accuracy of the Bi-LSTM and CNN models is relatively less satisfactory. This can be attributed to the fact that these models are typically applied to classification tasks rather than time-series prediction tasks [16,27].

**Table 1:** Model evaluation index.

	RMSE	MAE	MAPE	*R*^2^
CNN	0.2137	0.1472	37.70%	0.4409
GRU	0.0434	0.0315	6.78%	0.9721
Bi-LSTM	0.2663	0.2165	47.35%	0.1020
LSTM	0.0607	0.0490	12.73%	0.9457
BP	0.0619	0.0503	14.22%	0.9120
SVM	0.0719	0.0556	11.80%	0.9287

Table 2 shows the computational efficiency of the models. By comparing the running time, the traditional machine learning method is more efficient than the deep learning method. And the traditional machine learning method can also obtain good prediction accuracy based on Table 1. In addition, the prediction accuracy of traditional machine learning method can be enhanced by optimizing the parameters. Taking the BP neural network as an example, the network weight and threshold of BP model can be corrected through continuous training of data samples. The BP model with single-hidden-layer adopted in the experiment approximates the required “nonlinear function” through various hidden nodes, which is featured with extremely fast training. After parameter optimization, values of RSME, MAE, MAPE and *R*^2^ of BP neural network change from 0.0799, 0.0629, 13.40% and 0.9344 to 0.0578, 0.0433, 8.6% and 0.9539, respectively. However, as mentioned above, the models trained by BP and SVM models still need extra input data when predicting the value at a certain time, and the prediction accuracy decreases rapidly with the increase of the time steps [38,39]. In addition, existing researches indicate that, if the sample size and sample feature dimension increase, that is, with the increase of the complexity of the situation, pure mathematical models or prediction model taking traditional machine learning algorithm as the main support would hardly satisfy the practical demands [15,40].

**Table 2:** Model computational efficiency.

	Iterations	Training RMSE	Training Loss	Run time/s
CNN	120	0.12	0.01	5.58
GRU	200	0.28	0.04	10.78
Bi-LSTM	140	0.14	0.01	12.8
LSTM	300	0.28	0.04	18.03
BP	10	/	/	2.5
SVM	/	/	/	0.7

Compared with BP and SVM, the deep learning models of CNN, LSTM, Bi-LSTM and GRU require larger storage space for data increased with the accumulation of recurrent layer iterations and features due to their specific time connection characteristics. In view of this, the CNN, LSTM, Bi-LSTM and GRU models cost longer time in training. CNN model kernel is more efficient in extracting and calculating “memory” data based on convolution operation, and is easier for being used to carry out parallel operation, so that it can quickly obtain better convergence in model iterative computation. Compared with LSTM, GRU is characterized with optimized network structure, which greatly reduces the number of parameters required for training and improves the speed of model fitting. Therefore, as shown in Table 2, the runtime of the GRU model is 10.78 s, which is shorter than the 18.03 s required by the LSTM model.

Moreover, the training Loss and training RMSE shown in Table 2 indicate that, CNN and Bi-LSTM models have lower Loss and RMSE during the training stage, and can achieve rapid convergence. On the contrary, GRU and LSTM models require more iterations of training to obtain comparatively satisfactory convergence, for example: LSTM cannot achieve satisfactory convergence unless going through 300 iterations of training, and GRU shows convergence tendency after 200 iterations of training. On the other hand, it can be seen by comparing with data in Table 1 that, CNN and Bi-LSTM models have higher efficiency, but they can hardly achieve satisfactory prediction accuracy in time-series prediction.

Based on Table 1, Table 2, both the GRU and LSTM models yield higher prediction accuracy with fewer training iterations and shorter training time for time-series prediction of chloride transport in concrete. The GRU model is particularly efficient, as indicated by its *R*^2^ value of 0.9721 which is significantly higher than the *R*^2^ value of the LSTM model at 0.9457. Additionally, the GRU model outperforms the LSTM model in terms of RMSE, MAE, and MAPE values, all of which are notably lower in the GRU model. Furthermore, the computation time required for training the GRU model is also considerably shorter compared to that of the LSTM model.

**Fig. 12:**
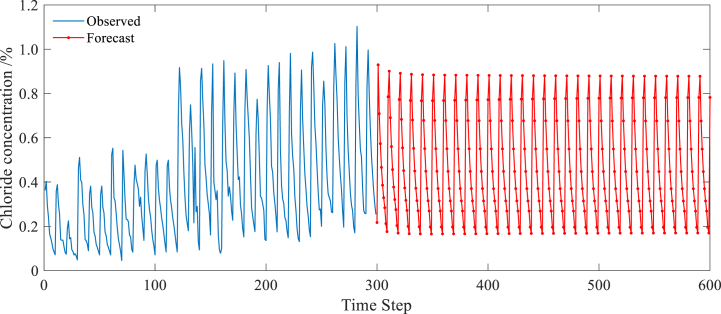
Chloride profiles predicted by GRU model.

The chloride profiles obtained through 300 steps of prediction using a GRU model are shown in Fig. 12. A comparison between Figs. 11 and 12 reveals that the chloride profiles predicted by an LSTM model are more accurate than those predicted by the GRU model in reflecting time-dependent chloride transport in concrete. The comparison also shows that, to simplify the model, GRU combines the forget gate and input gate into a single update gate, reducing the number of parameters by one-third compared to LSTM. This accelerates the training process and results in faster model convergence. However, during the training process, it is difficult to improve the GRU's efficiency in accurately extracting spatial-temporal features compared to LSTM. This is demonstrated by the significant decline in accuracy during dense and complex data oscillation periods—sacrificing accuracy for speed. As such, when predicting chloride profiles, the GRU model's prediction accuracy is not as good as that of the LSTM model when faced with complex and changing time-series characteristics.

## 4 Conclusions

Using field test data, this paper employs deep learning methods such as CNN, LSTM, GRU, and Bi-LSTM, as well as traditional machine learning methods like BP and SVM, to predict and analyze time-dependent chloride transport in concrete exposed to a coastal environment. Based on these analyses, the following conclusions have been drawn:

(1) The traditional machine learning methods of BP and SVM are characterized with high efficiency and satisfactory prediction accuracy. But the well-trained BP (or SVM) network can hardly be used to make further prediction if lacking external input data.
(2) CNN and Bi-LSTM models have low Loss and RMSE in the training stage, and the models can converge quickly. On the contrary, GRU and LSTM models need more iterations of training in order to maintain relatively good performance. But it is hard for CNN and Bi-LSTM models to obtain satisfactory prediction accuracy in predicting the chloride profiles.
(3) Both the GRU model and the LSTM model demonstrate higher prediction accuracy with fewer training iterations and shorter training time. The GRU model, in particular, exhibits higher efficiency. However, when it comes to further prediction tasks, the LSTM model tends to achieve higher prediction accuracy compared to the GRU model.
(4) The LSTM model significantly improves the prediction accuracy by optimizing the dropout layer, hidden unit, number of iterations, and initial learning rate. Values of MAE, MAPE, RMSE and *R*^2^ are 0.0271, 5.41%, 0.0357 and 0.9752, respectively, and the desirable chloride profiles of concrete specimens at 720d are predicted in the current work. It is feasible to predict the time-dependent chloride penetration in concrete exposed to coastal environment by LSTM model, and the short-term prediction results are accurate and credible.

## Author contribution statement

**Lingjie Wu**: Conceived and designed the experiments; Contributed reagents, materials, analysis tools or data; Wrote the paper. **Weiqiang Wang, Chenchi Jiang**: Performed the experiments; Analyzed and interpreted the data.

## Data availability statement

Data will be made available on request.

## Funding statement

This work was supported by the National Natural Science Foundation of China [grant numbers 52008317], the Natural Science Foundation of Zhejiang Province [grant numbers LQ20E080024] and the Science & Technology Association of Wenzhou City [grant numbers 2019KXX-KT5].

## Declaration of competing interest

The authors declare that they have no known competing financial interests or personal relationships that could have appeared to influence the work reported in this paper.
